# Enhancing lung transplantation with ECMO: a comprehensive review of mechanisms, outcomes, and future considerations

**DOI:** 10.1051/ject/2024023

**Published:** 2024-12-20

**Authors:** Salman Pervaiz Butt, Vivek Kakar, Salman Abdulaziz, Nabeel Razzaq, Yasir Saleem, Arun Kumar, Fazil Ashiq, Praveen Ghisulal, Aaron Thrush, Sadaf Malik, Mairead Griffin, Mahanoor Amir, Umar Khan, Ashal Salim, Zaid Zoumot, Izanee Mydin, Yazan Aljabery, Gopal Bhatnagar, Yusuf Bayrak, Andres Obeso, Usman Ahmed

**Affiliations:** 1 Interim Manager Perfusion Services, Heart Vascular and Thoracic Institute, Cleveland Clinic PO BOX 112412 Abu Dhabi United Arab Emirates; 2 Director ECMO Program, Critical Care Institute PO BOX 112412 Abu Dhabi United Arab Emirates; 3 Consultant of Cardiovascular Critical Care, Co-Chair of ECMO Task Force, Department of Health United Arab Emirates; 4 Clinical Perfusionist, Heart Vascular and Thoracic Institute, Cleveland Clinic PO BOX 112412 Abu Dhabi United Arab Emirates; 5 Perfusionist, All India Institute of Medical Sciences Sri Aurobindo Marg, Ansari Nagar New Delhi 110029 India; 6 Department Chair, Cardiothoracic Aesthesia, Anesthesiology Institute, Cleveland Clinic PO BOX 112412 Abu Dhabi United Arab Emirates; 7 Anesthesiology Physician, Anesthesiology Institute, Cleveland Clinic PO BOX 112412 Abu Dhabi United Arab Emirates; 8 Critical Care Associate Staff Physician, Critical Care Institute PO BOX 112412 Abu Dhabi United Arab Emirates; 9 Physical Therapist, Critical Care Institute PO BOX 112412 Abu Dhabi United Arab Emirates; 10 Physician Assistant, Critical Care Institute PO BOX 112412 Abu Dhabi United Arab Emirates; 11 Nurse Manager, Heart Vascular and Thoracic Institute, Cleveland Clinic PO BOX 112412 Abu Dhabi United Arab Emirates; 12 Physical Therapist, Shalimar Medical and Dental College Shalimar Link Road Lahore Punjab 54000 Pakistan; 13 Critical Care Consultant, Critical Care Institute PO BOX 112412 Abu Dhabi United Arab Emirates; 14 Charge Nurse, Critical Care Institute PO BOX 112412 Abu Dhabi United Arab Emirates; 15 Department Chair Pulmonology, Pulmonology Institute PO BOX 112412 Abu Dhabi United Arab Emirates; 16 Consultant Transplant Surgeon, The Newcastle Upon Tyne Hospitals NHS Foundation Trust, Freeman Hospital Freeman Road High Heaton NE7 7DN UK; 17 Associate Staff Physician, Heart Vascular and Thoracic Institute, Cleveland Clinic PO BOX 112412 Abu Dhabi United Arab Emirates; 18 Institute Chair, Heart Vascular and Thoracic Institute, Cleveland Clinic PO BOX 112412 Abu Dhabi United Arab Emirates; 19 Thoracic Physician, Heart Vascular and Thoracic Institute, Cleveland Clinic PO BOX 112412 Abu Dhabi United Arab Emirates; 20 Departmental Chair Thoracic Surgery, Heart Vascular and Thoracic Institute, Cleveland Clinic PO BOX 112412 Abu Dhabi United Arab Emirates

**Keywords:** Lung transplantation (LTx), ECMO (Extracorporeal Membrane Oxygenator), Primary graft dysfunction (PGD), Cardiopulmonary bypass (CPB)

## Abstract

*Background*: Lung transplantation (LTx) is a critical intervention for patients with end-stage lung disease. However, challenges such as donor organ scarcity and post-transplant complications significantly affect its success. Recent advancements in Extracorporeal Membrane Oxygenation (ECMO) have shown promise in improving the outcomes and expanding eligibility for LTx. *Methods*: A comprehensive literature review was conducted, focusing on studies that explore the use of ECMO in lung transplantation. A thorough search of relevant studies on ECMO and LTx was conducted using multiple scholarly databases and relevant keywords, resulting in 73 studies that met the inclusion criteria. Sources included peer-reviewed journals and clinical trial results, with emphasis on articles captured recent advancements in ECMO technology and techniques. *Results*: ECMO has been crucial in supporting patients before, during, and after LTx. It serves as a bridge to transplantation by maintaining pulmonary and circulatory stability in critically ill patients awaiting donor organs. ECMO also aids in the evaluation of marginal donor lungs and supports patients through acute post-transplant complications. Recent technological advancements have improved the safety and efficacy of ECMO, further solidifying its role in LTx. *Conclusion*: In conclusion, this review underscores ECMO's critical role in enhancing outcomes across all stages of lung transplantation. Its various configurations and strategies have shown promise in stabilizing critically ill patients and improving transplant success rates. Looking ahead, it’s important to gather more information about the long-term outcomes and potential complications associated with ECMO use. More research and data collection will help us understand the benefits and risks better, leading to improved decision-making and patient care in this field.

## Introduction

Lung Transplantation (LTx) is a life-saving intervention for patients with end-stage lung disease, offering a chance at improved quality of life and long-term survival. However, the success of LTx relies on factors like suitable donor organs, viable transplanted lungs, and effective management of post-transplant complications. Extracorporeal Membrane Oxygenation (ECMO) has emerged as a crucial therapy in LTx, revolutionizing patient care before, during, and after the transplant.

ECMO is based on the well-established cardiopulmonary bypass technology used in cardiac surgery. Nowadays circuit sizes are reduced and are often integrated with a sophisticated user interface and monitoring. The key components of an ECMO circuit include the cannulae for access and return of blood to the patient, often coated with biological materials to limit activation of the immune and coagulation pathways. A centrifugal pump is used to pump the blood around the circuit, often magnetically levitated, to limit trauma to blood cells. An oxygenator and gas blender for gas exchange, similar to the native lung, and hence also known as the “membrane lung”, with a much smaller machine lung surface area than the native lung is used. A heater-cooler to keep the blood traversing the extracorporeal circuit at a set temperature is also incorporated into the circuit.

The two common modalities of ECMO include veno-venous or VV ECMO and veno-arterial or VA ECMO and it has recently been proposed that a third entity called veno-pulmonary or VP ECMO be classified as a separate entity [1]. VV ECMO, as the name suggests, drains the blood and returns it to the patient’s venous system, and by that, it assists only in gas exchange, replacing and or supporting solely the lung function. It may be argued that by supplying oxygenated blood to the pulmonary circulation, it may reverse acute hypoxic pulmonary vasoconstriction and hence offload the right ventricle, which is noted to be strained in up to 50% of severe ARDS patients [2]. VA ECMO, on the other hand, returns the oxygenated blood to the patient’s arterial system, and hence it supports not only the gas exchange and lung function but by virtue of the centrifugal pump returns the blood with force into the aorta, thus supporting systemic circulation and heart function. VP ECMO returns the oxygenated blood to the pulmonary artery. VP ECMO offloads the right ventricle and supports both lung and right heart function. Recently, VP ECMO has been achieved using a single dual-lumen cannula [3]. All three modalities of ECMO namely VV, VA, and VP can be utilized in the perioperative period for lung transplant.

While ECMO has been utilized for long for respiratory and or cardiac failure, its use in lung transplantation has gained considerable attention recently for its potential to overcome many a transplant-related hurdle. The role of ECMO in LTx may be described as multi-faceted. It acts as a bridge to transplantation, supporting patients with severe respiratory failure while they await a suitable donor organ. By improving gas exchange and maintaining organ function preoperatively, ECMO increases the chance of successful transplantation and reduces deterioration in organ function during the waiting period. It also offers hope to several patients with end-stage disease who urgently need lung transplants but cannot be listed due to one or more contraindications. In some of these cases, patients can be salvaged with the support of ECMO, while the noted transplant barriers such as infection or other organ dysfunction are being optimised by excellent multidisciplinary care. Additionally, ECMO can assist during the transplantation surgery itself, allowing surgeons more time for the procedure and facilitating optimal organ preservation. In the immediate post-transplant period, complications including primary graft dysfunction, acute rejection, infection and right ventricular failure significantly impact patient outcomes. ECMO support post-lung transplant has shown promise in managing these complications by providing temporary respiratory and cardiac support, enabling the transplanted lungs to recover.

This comprehensive review aims to analyze existing research on ECMO in LTx, discussing its configurations, indications, contraindications, its utility as a bridge to transplantation, donor organ preservation, and post-transplant complications. The review will also explore advancements in ECMO technology that enhance its efficacy and safety providing valuable future considerations in the field of LTX [4].

## History of ECMO and lung transplantation

The roots of ECMO can be traced back to the 1950s when heart-lung bypass machines were first developed. Originally designed for open-heart surgeries, these machines allowed surgeons to divert blood from the heart and lungs, enabling them to operate on the heart while the machine took over the gas exchange and circulation of blood. In the following decades, medical professionals began exploring the potential of this technology for supporting patients with severe respiratory failure. This exploration led to the development of ECMO, a technique that involves extracting blood from the patient, oxygenating it externally, and then returning it to the body, effectively bypassing the lungs. The first successful utilization of ECMO to treat respiratory failure in a newborn took place in 1975. Since then, advances in technology and techniques, along with the establishment of specialized ECMO centres globally, have significantly enhanced the safety and applicability of ECMO. As a result, it has become a valuable option for patients experiencing respiratory distress, offering them renewed hope and improved outcomes [4, 5].

LTx, on the other hand, had its early roots in experimental attempts in the early 20th century, but significant progress was not achieved until the 1980 s. Fritz Derom performed the first successful single-lung transplant in 1981 where the patient survived for 10.5 months [6], Bruce Reitz followed with a successful double-lung transplant in 1986. These pioneering procedures paved the way for further advancements in LTx. One of the critical breakthroughs was the development of improved immunosuppressive medications to prevent graft rejection. The introduction of calcineurin inhibitors like cyclosporine and tacrolimus significantly improved long-term survival rates. Better surgical techniques complemented by improved organ preservation methods, donor-recipient matching, and post-operative care have not only contributed to improved outcomes but also resulted in the expansion of the eligibility criteria for LTx. Today, LTx is an established treatment option for end-stage lung diseases, and ongoing research aims to refine the procedure and further enhance outcomes [7].

**Figure 1:**
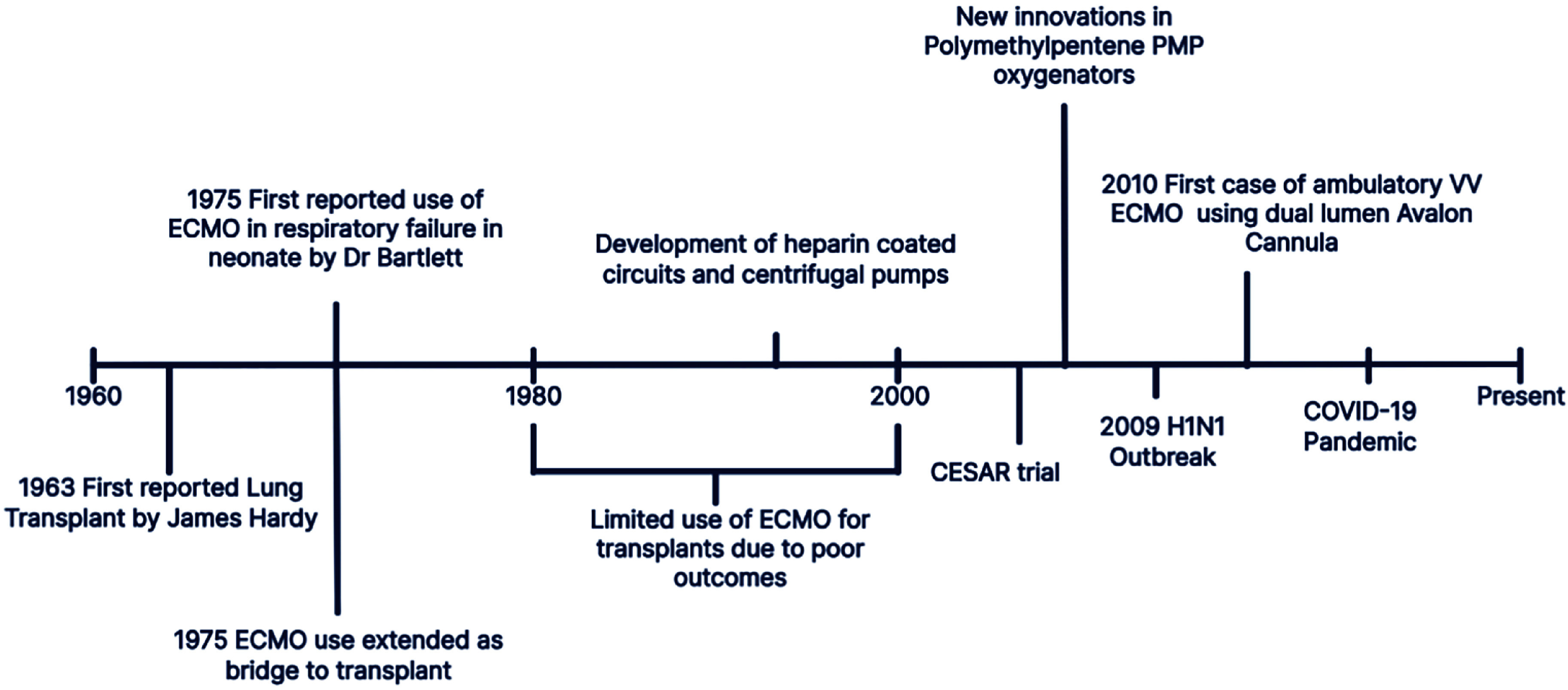
Depicts the milestones of ECMO, adapted and modified [4].

With the expansion of potential eligible recipients, the use of ECMO during the perioperative period was realized. The first case successfully bridged to LTx with ECMO was reported in 1977. The patient, who suffered from post-trauma respiratory failure, underwent bilateral LTx but survived only 10 days. In 1982, another patient was successfully bridged to a single LTx after 19 days on ECMO, with a short-term positive outcome in terms of survival. These early cases highlighted the potential of ECMO as a temporary respiratory support strategy for patients awaiting LTx [8]. Today, the use of ECMO in the care of LTx patients is not uncommon and its use is likely to grow further in coming years. Figure 1 shows a timeline of some key ECMO milestones [4].

## ECMO support in lung transplantation

### Lung preservation strategies

The field of LTx has witnessed significant advancements in preservation techniques over the past four decades. Initially, simple hypothermic immersion and topical cooling were used for lung preservation. However, the introduction of pulmonary artery flush combined with topical cooling became the standard technique due to its simplicity and effectiveness. Furthermore, the choice of preservation solution has evolved, with extracellular type solutions like Low-potassium dextran glucose (LPDG) and Celsior showing superior outcomes compared to intracellular type solutions.

Ex-vivo lung perfusion (EVLP) has gained popularity for assessing and preserving donor lungs, especially in cases of borderline lung suitability. Studies have indicated that storing lungs at 10 °C during cold static preservation may maintain mitochondrial health and preserve organ function compared to the conventional 4 °C storage temperature. Additionally, in-situ recruitment and assessment of atelectatic donor lungs prior to preservation have been found to improve post-transplant outcomes [9].

The study by Ali et al. explored a novel lung preservation technique involving alternating cycles of normothermic EVLP with cold static preservation at 10 °C. This approach successfully preserved donor lungs for a total of 3 days, maintaining stable lung function and histological structures. This new technique holds promise for multi-day lung preservation and may have significant implications for organ transplantation [10].

Ali et al. also conducted a clinical trial investigating the safety and potential benefits of extending cold static preservation times at 10 °C for LTx. The results indicated that longer preservation times were associated with a lower occurrence of grade 3 primary graft dysfunction at 72 h, suggesting that this strategy could enhance transplantation logistics and performance without adversely affecting outcomes [11].

Another approach to improving lung preservation involves adding nutrients and cytoprotective agents to preservation solutions. Studies using a cell culture model showed that maintaining physiological pH levels and utilizing phosphate-buffered media with colloid dextran 40 improved cell survival. Additionally, incorporating cytoprotective agents further enhanced the preservation process, offering the potential for designing more effective organ preservation solutions [12].

Overall, the advancements in lung preservation techniques have significantly improved the success and viability of LTx. The ongoing research and innovations in this field offer great promise for further optimizing lung preservation methods, leading to better outcomes for patients in need of life-saving lung transplants.

## Preoperative support

Bridge to LTx using ECMO is a critical strategy for patients with end-stage lung disease who require temporary respiratory support while awaiting a suitable donor organ. Several studies on outcomes and challenges associated with ECMO as a bridge therapy for LTx, provide valuable insights as well as comparable results.

Hunt et al. explored the outcomes of COVID-19 patients bridged with ECMO before LTx. Despite higher rates of post-operative complications, ECMO patients did not show significant differences in survival rates at 1 and 6 months post-transplant compared to non-ECMO patients. These findings suggest that ECMO can be safely used as a bridge to LTx in carefully selected COVID-19 patients with respiratory failure, even with prolonged support [13].

Sainathan et al. found in paediatric patients aged 12–18 years that the patients bridged to LTx with ECMO had comparable one-year and three-year survival rates to non-bridged patients, despite higher acuity, indicating that ECMO offers acceptable operative mortality and long-term survival rates in this age group [14].

Ko et al. conducted a multicentre prospective observational study, again finding comparable outcomes in patients bridged with ECMO to LTx compared to the control group, with no significant differences in post-transplant complications and hospital mortality [15].

Fischer et al. investigated the utilization of the pumpless interventional lung assist device called NovaLung as a bridge to LTx for patients with severe ventilation-refractory hypercapnia. The findings of the study revealed the remarkable effectiveness of NovaLung in enhancing oxygenation and acid-base balance, resulting in successful bridging and an impressive 80% one-year survival rate after transplantation [16].

Oh et al., however, evaluated the impact of ECMO as a bridge to LTx (BTT) on post-transplant outcomes. Although the bridge-ECMO group showed comparable 1-year and 5-year post-transplant survival rates to the non-BTT group, long-term ECMO bridging (≥14 days) was identified as an independent risk factor for 1-year post-transplant mortality demonstrating potential risks [17]. In addition Atchade et al. highlighted the impact of ECMO as a bridge to LTx on bronchial anastomotic dehiscence (BAD) occurrence. ECMO support was identified as an independent risk factor for BAD, emphasizing the need for close monitoring and vigilance in high-risk recipients during the early post-operative period [18].

A retrospective study done by Rando et al. using the United Network for Organ Sharing (UNOS) database, looked at 40,866 patients over the age of 19 years, of whom 1387 (3.4%) were on ECMO and 39,479 (96.6%) had no ECMO. Average age and initial Lung Allocation Score increased significantly during the study period in both cohorts but occurred slower in the ECMO population. The hazard of death was significantly lower in years 2015–2019 for both the ECMO and non-ECMO cohorts with an adjusted hazards ratio (aHR) of 0.59, 95% confidence interval (CI) 0.37–0.96 and aHR of 0.74, 95% CI 0.70–0.79), when compared to the early years 2000–2004. The study highlighted that post-transplantation survival was higher for patients bridged to transplantation with ECMO, and demonstrates ongoing improvement despite cannulation of progressively older and sicker patients [19].

The development of risk assessment tools such as the STABLE score by Habertheuer et al. may contribute to some improved outcomes. The score provides a valuable risk assessment tool for predicting in-hospital mortality in patients requiring ECMO as a bridge to LTx. This score can aid clinicians in stratifying risk and making informed decisions to potentially improve post-transplant outcomes [20].

In another study on the use of ECLS as a bridge to LTx in a larger cohort of patients, out of 1111 lung transplants performed over a 10-year period, 71 adults received ECLS as a bridging therapy. The majority of patients survived LTx, with an overall survival rate of 89% demonstrating its use. However, the study highlighted an important distinction between patients bridged to first lung transplant and those bridged to re-transplantation. Patients bridged to re-transplantation had significantly shorter survival compared to those bridged to first lung transplant. This suggests that caution should be exercised when considering ECLS as a bridge to re-transplantation although this is not as common on the whole [21].

Collectively, these studies demonstrate the significance of ECMO as a bridge to LTx, offering option to patients with severe respiratory failure. ECMO provides temporary respiratory support, stabilizing patients’ conditions and preserving vital organ function while awaiting a donor organ. Careful patient selection and close monitoring of potential complications are essential to ensure successful bridging and optimal post-transplant outcomes. In patients with chronic respiratory failure, ECMO as a bridge helps to stabilize the potential recipient in multiple ways as discussed. In patients with prolonged acute respiratory failure secondary to primary pneumonia, long ECMO runs have provided important insights for lung recovery in acute illness.

Patients with advanced age, those with organ failures other than lung, severe deconditioning secondary to malnutrition, and neuromyopathy are likely to have poor outcomes and should not be routinely considered for ECMO as a bridge to LTx. As the understanding of ECMO’s role in LTx continues to evolve, these barriers would reduce and more critically ill patients could be considered for ECMO as a bridge to LTx in coming years.

ECMO has even been shown to be used as a treatment to recovery to avoid lung transplantation. There are multiple reports of patients recovering after ECMO when the lung has been given time to repair. De Walue et al. did a retrospective study [22] looking at the maximum amount of time under VV ECMO where pulmonary recovery remains possible. 14 patients who were on VV ECMO for more than 50 days with COVID-19 and very severe ARDS were included in the study. Recovery was reported in 10 patients, with one patient on ECMO for 151 days, deeming this a good treatment and avoiding lung transplantation. Unnecessary transplants could thus be avoided in at least some patients with acute respiratory failure.

Currently, research is being done to develop treatments to enhance lung regeneration and repair. Managing the inflammatory response and infection of pathogens seems to be key to these improvements [23]. Stem cell therapy is also emerging, where endogenous lung progenitors, induced pluripotent stem cells, and embryonic cells may be the way forward in introducing truly regenerative lung cells for the future [24].

## Intraoperative support

### Anesthesia support

Anesthesia management for lung transplants with veno-arterial extracorporeal membrane oxygenation (VA ECMO) involves careful coordination between the anesthesia team, the transplant surgeons, and the ECMO specialists.

A preoperative evaluation of the patient’s medical history including the indication for transplant as well as a physical examination is carried out. A careful review of laboratory parameters such as full blood count, coagulation profile, metabolic profile as well inflammatory markers, echocardiogram, CT scan, and right heart catheterisation measurements is done. An anesthetic plan is made in discussion with the surgeon as well as the perfusionist. Anesthetic induction is carried out in a gentle manner minimizing hemodynamic fluctuations and keeping in mind the degree of pulmonary hypertension and right ventricular function. Continuous monitoring of arterial blood pressure, central venous pressure, cardiac output, and cerebral oximetry along with arterial blood gases and electrolytes form the corner stone of anesthetic management. Close coordination with the perfusionist is key to managing anticoagulation on ECMO. A fine balance must be kept in mind to balance the risk of clot in the circuit versus excessive bleeding and thromboelastography or thromboelastometry may be used as a guidance.

The ventilation strategy may vary depending on the patient’s lung condition and the phase of the transplant procedure. Lung protective ventilation strategies are typically employed to minimize ventilator-associated lung injury. Following the procedure, continuous monitoring in the intensive care unit (ICU) with close collaboration between the anesthesia team, transplant surgeons, and critical care specialists is done where management of pain, sedation, and ventilatory support in the postoperative period is looked at. Gradual weaning of ECMO support based on the patient’s clinical status and lung function is the goal.

Utmost vigilance is called for to avoid and address promptly complications such as bleeding, thrombosis, and hemolysis associated with ECMO. Overall, anesthesia management for lung transplant with VA ECMO requires a multidisciplinary approach, with close communication and coordination among all members of the healthcare team to optimize patient outcomes [25–27].

### Intraoperative ECMO

ECMO plays a crucial role in LTx by providing temporary cardiopulmonary support intraoperatively. The use of VA ECMO intraoperatively mitigates the need for full cardiopulmonary bypass and thereby avoids all potential complications of cardiopulmonary bypass, such as less activation of coagulation cascade, thrombocytopenia and systemic inflammatory response syndrome leading to severe vasodilatory shock. The use of ECMO during LTx still presents potential challenges, including the risk of bleeding, thrombosis, infection, and organ dysfunction. These risks highlight the importance of careful monitoring and specialized care throughout the surgical procedure to mitigate complications. Despite these challenges, studies have shown that ECMO support during LTx can yield favourable outcomes.

Lus et al. emphasized the significant improvements in perioperative cardiopulmonary support for LTx through the use of extracorporeal life support (ECLS). Enhanced patient management, multidisciplinary collaboration, and standardized ECLS protocols have contributed to excellent outcomes in high-volume transplant centres. Although ECMO supported patients may experience a higher prevalence of complications, there may be no significant difference in long-term graft function compared to non-supported patients. It is worth noting that the evidence supporting ECLS in LTx is primarily based on real-world experience rather than randomized controlled trials [28].

The outcomes of LTx with and without ECMO were examined in a study involving 48 lung transplants. While the 30-day mortality rate was higher in patients with ECMO, the difference was not statistically significant. The study highlighted a successful ECMO weaning rate and indicated that ECMO was an effective adjunctive support during surgery for critically ill lung transplant recipients [29].

Routine intraoperative ECMO has shown promise in improving primary graft function and mid-term outcomes in bilateral LTx, as demonstrated by Hoetzenecker et al. [30]. Patients who underwent bilateral LTx with ECMO support exhibited low rates of primary graft dysfunction, favourable extubation times, and survival rates. These findings support the recommendation for routine intraoperative ECMO in bilateral LTx to enhance patient outcomes [31].

Intraoperative extracorporeal assistance in lung transplant has also been reviewed by Ruszel et al., focusing on 77 lung transplant cases. The study highlighted that central ECMO had higher survival rates compared to peripheral ECMO or cardiopulmonary bypass (CPB). However, patients with support devices were more prone to acute kidney injury and thromboembolic complications. The study recommended the preference of central ECMO over peripheral cannulation or CPB during LTx [32].

In addition to ECMO support as described above, Taka et al. investigated the outcomes of lung transplant surgery using cardiopulmonary bypass (CPB) and a protective allograft reperfusion strategy. The results indicated that CPB, along with the protective reperfusion approach, did not result in life-threatening graft complications within the first 72 hours post-surgery. The survival rates for patients who received lungs from extended criteria donors (ECD) were favourable, comparable to those who received lungs from standard criteria donors (SCD). This suggests that using CPB with a protective reperfusion strategy does not negatively impact survival outcomes in lung transplant surgery with extended criteria donors [29].

In conclusion, ECMO serves as a crucial support system during the LTx procedure, providing temporary cardiopulmonary support.

### Hybrid circuit

**Figure 2:**
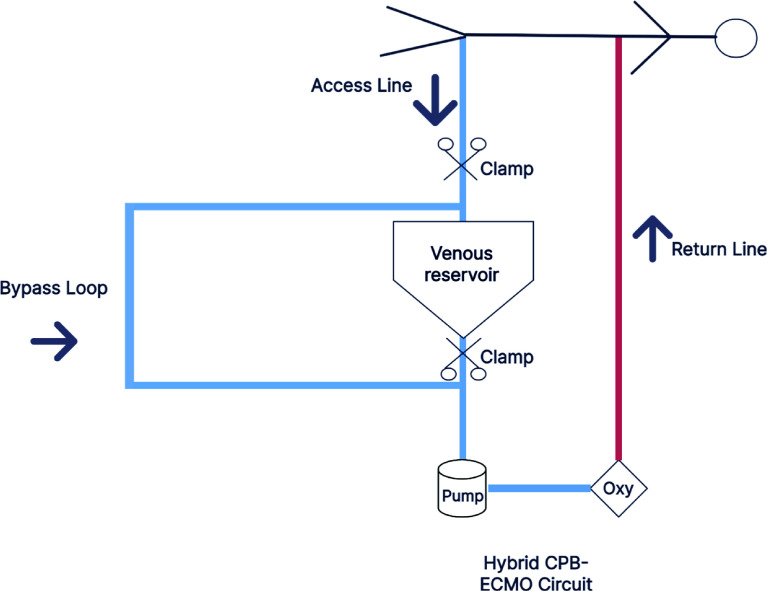
Illustration of hybrid circuit for LTx.

Some centres are implementing VA ECMO with a hybrid ECMO-CPB circuit during lung transplants. An example of this from our centre can be seen in Figure 2.

This set up has all the benefits of a closed ECMO circuit as mentioned previously, but in addition, using the circuit without the venous reservoir reduces the priming volume and haemodilution, thus limiting the need for perioperative transfusions. In addition, the inflammatory response is reduced as a result of less artificial surface interacting with the patient’s blood, and there is less blood-air interface due to the lack of a reservoir. The benefit of this has been extensively studied where in full CPB, the inflammatory response seen is higher with release of cytokines IL-1β, IL-6, IL-8, and TNF-α with vasodilating properties, through the expression of nitric oxide (NO) synthase. This results in a decrease in vascular resistance and higher usage of vasopressors. A lower heparin dose is also possible with the ECMO circuit, especially if the circuit used is heparin-coated. The overall benefit of using ECMO is reduced organ damage and post-operative complications [33–37].

If the need however arises that full CPB is needed due to excessive bleeding, or haemodynamic instability, the circuit can easily be transitioned by clamping out the ECMO bypass loop and diverting blood through the reservoir. Pump suckers and vacuum assist can also be used in this setting and the circuit is fully set up in case the heart needs to be operated on and there is a need for cardioplegia.

The circuit components itself are a centrifugal pump which has been shown to be beneficial in prolonged cases [38] and in this case, a polymethylpentene (PMP) oxygenator, which has the added benefit of being able to be incorporated in the ECMO circuit postoperatively if needed.

During the surgery in our centre, ACT levels are kept above 200 seconds when using the hybrid ECMO circuit. Volume and systemic vascular resistance are managed by the anaesthetist to maintain the preload and afterload of the patient and thus helping maintain the full flows of the closed system.

## Postoperative support

Primary graft dysfunction (PGD) is a severe complication following LTx that can lead to respiratory failure within the first 72 hours. In such critical cases, ECMO plays a crucial role in providing temporary respiratory and circulatory support. By reducing strain on the graft and allowing the damaged lungs to rest and heal, ECMO increases the chances of graft recovery. It also serves as a valuable tool for assessing the viability of the transplanted lung and making informed decisions on re-transplantation if necessary. A centre-specific study has also been conducted to assess the impact of ECMO following LTx, with a focus on patient outcomes. The findings unveiled several factors associated with the need for post-transplant ECMO, including vascular disease as the cause of transplantation, advanced donor age, and the requirement for cardiopulmonary bypass during the transplant procedure [39].

Hunt reviewed the risk factors and management strategies for PGD. Supportive care is crucial in managing PGD, and early initiation of ECMO has been shown to improve outcomes in certain patients. The study also discussed the potential benefits of ex-vivo lung perfusion platforms in reducing the risk of PGD and increasing lung transplant volume, although the impact on survival requires further investigation [40].

Van Slambrouck et al. provided a multidimensional understanding of PGD at various levels, including clinical, physiological, radiological, histological, and cellular. This comprehensive approach enhances our knowledge of acute lung failure in lung transplant recipients and provides insights for future therapeutic strategies [41]. Harano et al. examined the predictors and outcomes of early mortality in post-lung transplant patients who required ECMO for PGD. The study found that earlier recognition of PGD and prompt initiation of ECMO may improve outcomes in this patient population. Delayed initiation of ECMO after transplantation was associated with higher in-house and 3-year mortality rates [42].

These studies highlight the critical role of ECMO in managing severe PGD after LTx and heart transplantation. Early initiation of ECMO has been associated with improved outcomes, while delayed initiation or the use of VA ECMO for grade 3 PGD may increase the risk of mortality. Careful patient selection, timely recognition of PGD, and prompt initiation of appropriate ECMO support are crucial factors in optimizing outcomes in these high-risk patients.

### ECMO configurations

The integration of ECMO has become an important component of LTx, offering diverse applications throughout various stages of the procedure. VV ECMO is commonly employed for patients experiencing hypercapnic or hypoxic respiratory failure, while VA ECMO may be necessary for those facing hemodynamic instability. During the intra-operative phase, VV ECMO can be either continued or switched to VA ECMO as required. Recent investigations have delved into the potential advantages of routine intra-operative ECMO, such as enhancing graft function, reducing primary graft dysfunction, and mitigating the effects of ischemia-reperfusion injury during the postoperative period [8, 43].

Takahashi et al. assessed the outcomes of patients with grade 3 PGD who required ECMO support after LTx. The study found that the use of venoarterial (VA) ECMO for grade 3 PGD was associated with increased mortality compared to venovenous (VV) ECMO or non-ECMO treatment. This suggests that VA ECMO treatment for grade 3 PGD is a significant risk factor for mortality [44].

The impact of different cannulation methods for V-V ECMO was examined in a retrospective, multicentre study, in patients with severe respiratory failure caused by COVID-19 undergoing V-V ECMO. The study involved 435 adult patients from 17 centres, divided into three groups based on cannulation method: dual-site cannulation, cannula in the pulmonary artery, and cannula in the inferior vena cava. The findings indicated that using a single dual-lumen cannula positioned in the pulmonary artery via the internal jugular vein was associated with lower mortality rates compared to the other methods. However, it is important to consider the limitations of the study, and further research is necessary to validate these results [45]. Another study by Parker et al. compared atrio-femoral and femoro-atrial cannulation methods in V-V ECMO. Atrio-femoral cannulation showed 13.5% higher recirculation under ideal conditions, with flow patterns resembling normal physiology. Femoro-atrial cannulation led to multiple vortices and increased turbulent kinetic energy at higher flow rates. Factors such as occlusion of side holes and inferior vena cava inflow affected recirculation. The choice of cannulation should consider drainage issues, and the proximity of the cannula tip did not significantly impact recirculation [46].

Ruszel et al. in their study found that among patients undergoing LTx, approximately half required assistance with ECMO or cardiopulmonary bypass. Central ECMO was associated with higher survival rates compared to no support, peripheral ECMO, or cardiopulmonary bypass. However, the use of support devices increases the risk of acute kidney injury and thromboembolic complications. The study recommended favouring central ECMO over other methods and discontinuing cardiopulmonary bypass during LTx [32].

### ECMO strategies

Kim et al. found that awake patients on ECMO had better postoperative outcomes compared to non-awake patients. The awake group experienced shorter stays in the intensive care unit, longer periods free from ventilator support, higher gait ability after transplantation, and improved lung function at 6 months and 1 year. Furthermore, the awake group had significantly lower mortality rates at 6 months and 1 year compared to the non-awake group. These findings suggest that the awake ECMO strategy may be beneficial for patients with end-stage lung disease awaiting transplantation [47].

Patients with end stage lung disease often succumb to intubation and the common resulting complications including critical illness polyneuropathy, making it difficult to maintain the physical function that is required for impending lung transplantation. Because limited functional status or poor rehabilitation potential are absolute contraindications to lung transplantation, efforts to minimize loss of strength and function are crucial [48].

Active rehabilitation and ambulation while on ECMO are proposed for carefully selected patients, and should be delivered by highly trained multidisciplinary teams typically led by physical therapists [49–52]. Published procedures and algorithms for patient selection and progressive mobilization, as well as ECMO configurations, are available to facilitate the uptake of this practice [52–54].

Interest in awake and ambulatory ECMO is evident in early published descriptions of small cohorts of bridge to transplant candidates [49, 55–57], albeit lacking detailed descriptions of physical therapy interventions.

Subsequent efforts to establish the safety and feasibility of awake and ambulatory ECMO in larger retrospective cohorts were successful, with low rates of adverse events [50, 58] and minimal impact on physiologic parameters even in intensive rehabilitation intervention groups [59]. Study inclusions have widened to incorporate patients with femoral cannulation sites, also with positive safety profiles [51, 60].

Recent evidence demonstrates the potential impact of awake and ambulatory ECMO – including improved function, clinical outcomes, and survival [61–64]. Patients cannulated for transplantation are observed to achieve earlier and greater mobility compared to non-transplant patients [60, 64]. Reduced total hospital costs and post-transplant ICU costs of an ambulatory cohort supported on ECMO further promotes this as an economically superior management strategy [65]. However, high-quality investigations into exercise training protocols and elucidating short- and long-term effects remain essential [66, 67].

### ECMO complications

**Figure 3:**
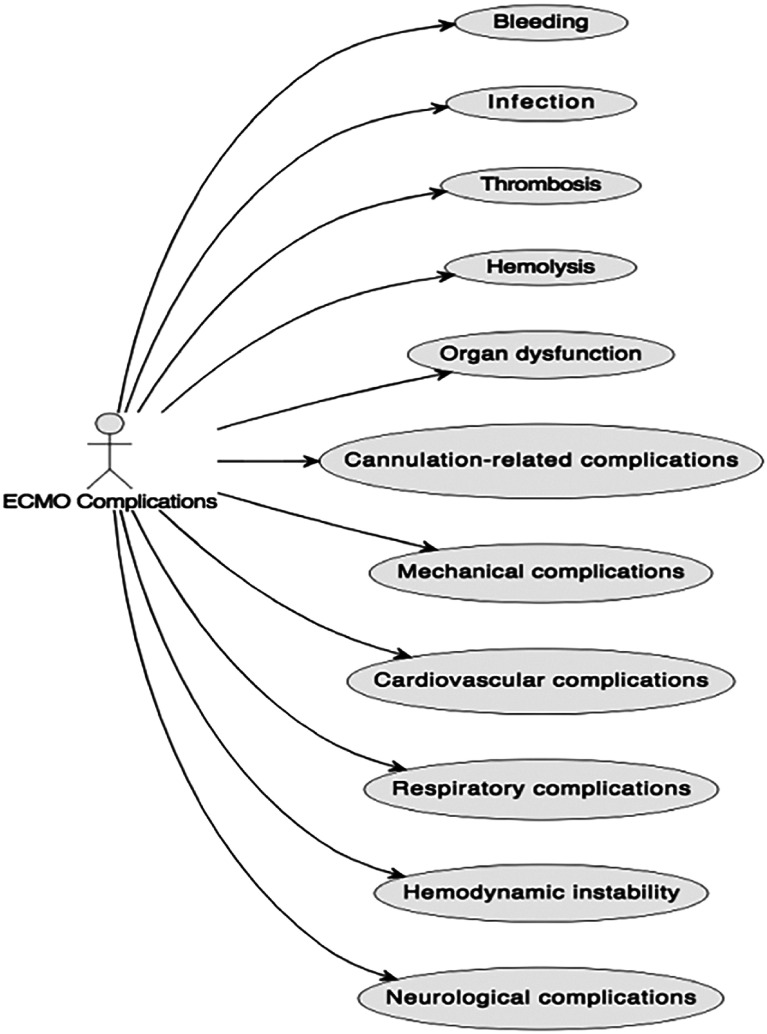
Illustration of major ECMO complications.

During LTx, the use of ECMO can provide vital support to the patient’s heart and lungs. However, this technique is not without potential complications. Some of the risks associated with ECMO in LTx include bleeding, infection, hemolysis, organ dysfunction, thromboembolism, cannula-related complications, neurological issues, barotrauma, and challenges specific to veno-arterial ECMO. These complications can arise from factors such as anticoagulation, cannula insertion, stress on other organs, and the mechanical forces within the ECMO circuit. Achieving hemostasis when bleeding occurs (which can occur ~10–30% of patients on ECMO), particularly with hemoptysis, can prove particularly problematic and occasionally persists even when anticoagulation is withdrawn. The medical team closely monitors patients for these complications, striving to strike a balance between the potential benefits and risks of ECMO in each individual case [68]. Figure 3 shows a list of ECMO complications.

### ECMO circuit related complications

ECMO circuit-related complications can arise during therapy. These complications include circuit occlusion or clotting, circuit leaks, pump malfunction, oxygenator dysfunction, air embolism, infection or sepsis, hemolysis, and circuit disconnection or accidental decannulation. Circuit occlusion or clotting can impede blood flow, requiring trouble shooting measures such as checking for obstructions and adjusting anticoagulation. Circuit leaks may result in air or blood ingress, necessitating thorough inspection and prompt resolution. Pump malfunction can disrupt adequate flow, and oxygenator dysfunction may affect gas exchange efficiency. Air embolism poses a risk and requires meticulous de-airing procedures. Infection or sepsis can develop, necessitating stringent aseptic techniques and appropriate antibiotic therapy. Hemolysis may occur, requiring monitoring and assessment of potential causes. Circuit disconnection or accidental decannulation demands immediate action to ensure patient safety and circuit reconnection. Proactive management and troubleshooting are crucial to address these complications and ensure optimal ECMO support [69–72].

## Future considerations

Based on our institutional experience the way to advance the use of ECMO in pre, intra, and post-lung transplant settings, several key considerations should be addressed. These include:

- Refining patient selection criteria to identify optimal candidates for ECMO support, based on predictive indicators and thresholds.
- Optimizing ECMO management protocols, including standardized initiation, weaning, and troubleshooting processes, cannulation techniques, circuit configurations, anticoagulation strategies, and monitoring parameters.
- Advancing ECMO technology, such as developing more efficient and user-friendly systems, improved oxygenators, centrifugal pumps, circuit materials, and integrated monitoring and remote management capabilities.
- Evaluating long-term outcomes of ECMO-supported lung transplants to assess graft function, quality of life, and potential complications beyond the immediate post-transplant period.
- Conducting comparative studies to determine the effectiveness of ECMO compared to alternative interventions or strategies through randomized controlled trials and large-scale observational studies.
- Assessing the cost-effectiveness of ECMO in lung transplantation, including equipment, personnel, hospitalization, and long-term care costs, to optimize resource allocation.
- Promoting multidisciplinary collaboration among transplant surgeons, anaesthetists, pulmonologists, intensivists, perfusionists, and other healthcare professionals for comprehensive and coordinated care.
- Enhancing education and training programs to ensure competent healthcare professionals proficient in ECMO management, through comprehensive training, sharing best practices, and standardized educational curricula [73].

Addressing these considerations will contribute to further advancements in ECMO utilization in lung transplantation, improving patient outcomes and guiding future research and practice.

## Conclusion

In conclusion, this review emphasizes the significant role of ECMO in improving outcomes throughout the pre-, intra, and post-lung transplant phases. The utilization of different configurations and strategies has demonstrated promising results in stabilizing critically ill patients, enhancing transplant outcomes, and increasing the likelihood of successful lung transplantation. However, there are important future considerations that must be addressed to further advance the use of ECMO in these transplant settings.

A crucial area for improvement is the need for more comprehensive knowledge regarding long-term outcomes and potential complications beyond the immediate post-transplant period. By conducting further research and collecting data in this domain, we can enhance our understanding of the benefits and risks associated with ECMO in lung transplant recipients. This knowledge will facilitate informed decision-making and optimized patient care in this rapidly evolving field.

## Data Availability

The research data are available on request from the authors.
